# Dental eruption in afrotherian mammals

**DOI:** 10.1186/1741-7007-6-14

**Published:** 2008-03-18

**Authors:** Robert J Asher, Thomas Lehmann

**Affiliations:** 1 Department of Zoology, University of Cambridge, Downing St., Cambridge CB2 3EJ, UK; 2 Museum für Naturkunde, Humboldt Universität zu Berlin, Abteilung Forschung, Invalidenstr. 43 10115 Berlin, Germany

## Abstract

**Background:**

Afrotheria comprises a newly recognized clade of mammals with strong molecular evidence for its monophyly. In contrast, morphological data uniting its diverse constituents, including elephants, sea cows, hyraxes, aardvarks, sengis, tenrecs and golden moles, have been difficult to identify. Here, we suggest relatively late eruption of the permanent dentition as a shared characteristic of afrotherian mammals. This characteristic and other features (such as vertebral anomalies and testicondy) recall the phenotype of a human genetic pathology (cleidocranial dysplasia), correlations with which have not been explored previously in the context of character evolution within the recently established phylogeny of living mammalian clades.

**Results:**

Although data on the absolute timing of eruption in sengis, golden moles and tenrecs are still unknown, craniometric comparisons for ontogenetic series of these taxa show that considerable skull growth takes place prior to the complete eruption of the permanent cheek teeth. Specimens showing less than half (sengis, golden moles) or two-thirds (tenrecs, hyraxes) of their permanent cheek teeth reach or exceed the median jaw length of conspecifics with a complete dentition. With few exceptions, afrotherians are closer to median adult jaw length with fewer erupted, permanent cheek teeth than comparable stages of non-afrotherians. Manatees (but not dugongs), elephants and hyraxes with known age data show eruption of permanent teeth late in ontogeny relative to other mammals. While the occurrence of delayed eruption, vertebral anomalies and other potential afrotherian synapomorphies resemble some symptoms of a human genetic pathology, these characteristics do not appear to covary significantly among mammalian clades.

**Conclusion:**

Morphological characteristics shared by such physically disparate animals such as elephants and golden moles are not easy to recognize, but are now known to include late eruption of permanent teeth, in addition to vertebral anomalies, testicondy and other features. Awareness of their possible genetic correlates promises insight into the developmental basis of shared morphological features of afrotherians and other vertebrates.

## Background

Only within the last decade has it become apparent that Afrotheria, consisting of elephants (Proboscidea), sea cows (Sirenia), hyraxes (Hyracoidea), aardvarks (Tubulidentata), sengis (Macroscelididae), tenrecs (Tenrecidae) and golden moles (Chrysochloridae), comprises a natural assemblage of mammals descended from a single common ancestor [1]. Morphological features shared by these animals, particularly between the insectivoran- and ungulate-grade afrotherians, have been difficult to identify. However, several interesting similarities have recently become apparent, including non-descent of the male gonads [2], morphology of the placenta [3], variable vertebral count [4,5], a concave cotylar facet of the astragalus [6], calcaneo-navicular contact and possibly other features that are dependent on topology and assumptions of character optimization [7]. Although it was not recognized as such at the time, Wilhelm Leche [8] observed an additional similarity in 1907:

"The Centetidae [= Tenrecidae] and Chrysochloridae contrast with nearly all other insectivorans, and from the large majority of other extant mammals, in that eruption of the permanent dentition occurs in a very late period of life, that is, after the individual is fully grown and sexually mature" (translated from [8], pp. 38–39).

Leche could not have known about the genetic evidence in favor of Afrotheria. He interpreted this feature in tenrecs and golden moles as primitive for therian mammals, and used it as a basis to develop his theory that the deciduous dentition is morphologically more indicative of evolutionary relationships than the permanent dentition. While his observations regarding deciduous teeth seem not to have had great resonance since 1907 (or to have been thoroughly tested), delayed dental eruption in African insectivorans may be very significant. Eruption of permanent teeth well past sexual maturity is not common among mammals, except in elephants [9], sea cows [10] and hyraxes [11]. The remaining afrotherian clade, Tubulidentata, does not erupt a functional, deciduous dentition. Aardvarks are ontogenetically not well documented, with the last moderately comprehensive study of their dental development having taken place in 1934 (see [12]). We briefly discuss this taxon below, but defer a fuller treatment to a separate analysis (Lehmann, Notes on the ontogeny of the aardvark (*Orycteropus afer*), submitted).

Considerable variation occurs across mammals in the number and extent of replacement among incisors, canines, premolars and in the number of permanent molars [13]. Less variable is the number of teeth throughout any single mammalian lifetime and the age by which the permanent dentition is fully erupted. Nearly all mammals have only one or two generations, both limited in number of teeth. Only *Trichechus *(Sirenia) and *Petrogale concinna *(Diprotodontia) continually erupt supernumerary molars throughout their lifetime [10,14]. Complete eruption of the permanent dentition in most mammals occurs by sexual maturity [15]. Slight delays are known in primates and ungulate-grade mammals [15,16]. However, afrotherians such as elephants, sea cows and hyraxes may spend well over half of their lifespan without a completely erupted permanent dentition [9-11].

In this paper, we suggest that this pattern also characterizes small afrotherians (i.e., sengis, tenrecs and golden moles) and that delayed dental eruption comprises a shared feature of Afrotheria. As data on dental ontogeny are still lacking for small afrotherians, we evaluate metrics of size and tooth ontogeny designed to test Leche's still-unquantified observation that these taxa exhibit late eruption of the permanent dentition.

In addition, we note a fascinating similarity between the pattern of dental ontogeny in afrotherians and one of the most characteristic symptoms of a human genetic pathology: cleidocranial dysplasia (CCD) [17]. CCD is an autosomal dominant disorder resulting from mutations to the transcription factor *Runx2 *[18,19] which contributes to regulation of the development of bone and teeth [20]. Humans with CCD may exhibit not only delayed eruption of permanent teeth, but also supernumerary teeth, hemivertebrae, non-descended testes [21], incompletely ossified clavicles and cranial dermal bones, among other anomalies [22,23]. While the extent and form of these pathologies vary considerably across individuals, Mundlos [23] noted that delayed eruption of permanent teeth "is a relatively constant finding. Dental disability begins in late youth with the progressive morbidity and loss of the deciduous dentition. Many patients remember living 'without teeth' for some years until the permanent teeth eventually erupted" (from [23], p. 178). Other pathologies resulting in delayed dental eruption relate to nutritional defects, thyroid insufficiency, irradiation or trauma [24]. However, to the best of our knowledge, other genetic conditions do not consistently result in this phenotype.

CCD-like characters are known to occur across a number of mammalian clades, not only afrotherians. In addition to investigating the extent of delayed eruption in afrotherians, we also present a preliminary test of the hypothesis that delayed eruption, vertebral anomalies, reduced clavicles, incomplete suture of cranial bones and testicondy covary across mammals, as would be expected if they were the result of a conserved genetic and/or developmental program.

## Results

### Dental eruption relative to skull size

**Figure 1:**
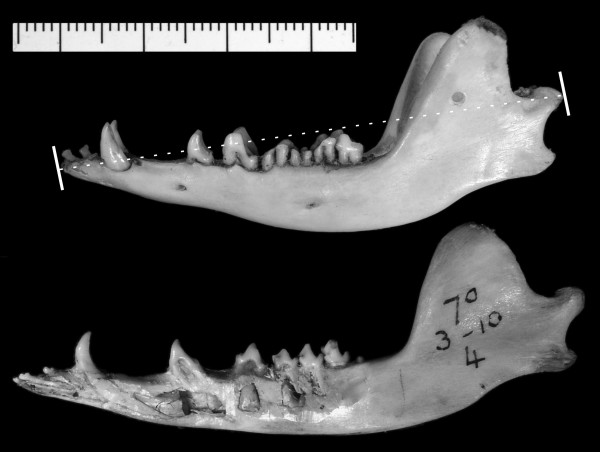
**Jaws of *Tenrec ecaudatus *in lateral view**. These images demonstrate how adult size in an afrotherian may be reached in the absence of many permanent cheek tooth loci. The old individual above has its complete permanent dentition erupted, showing p4-m2 worn down to their roots (UMZC H5431J, image reversed). The individual below is larger, but retains its deciduous canine through dp4; their permanent successors plus m3 are still unerupted within the dentary (BMNH 70.3.10.4). Scale bar (in millimeters) applies to both specimens. The dotted line from the condyle to symphysis on the top specimen represents measure taken of maximum jaw length, quantified in Figure 2.

Absolute age data for dental eruption in tenrecs, golden moles and sengis are not yet available in the literature. As an alternative, we compared the proportion of erupted permanent cheek teeth with metrics of skull size. We expect that if these taxa show delayed eruption of the permanent dentition, then some or all of their permanent teeth should finish erupting after the completion of growth, leading to specimens at or near adult size without a full complement of permanent teeth. Anecdotal observations of this nature (Figure 1) are in part what led Leche [8] to draw the conclusion that tenrecs and golden moles show delayed eruption.

**Figure 2:**
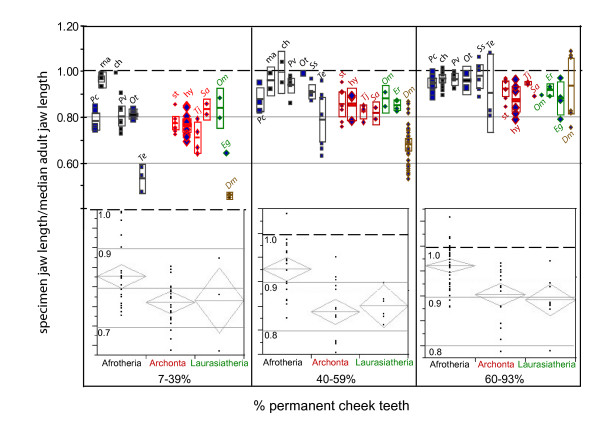
**Proportion of median adult jaw length (*y*-axis) for mammals with incompletely erupted cheek teeth (*x*-axis)**. Comparisons of individual taxa are shown above and between high-level clades below. Thick dotted horizontal lines (at 1.0) indicate median adult jaw length. For each stage of incomplete cheek tooth eruption (roughly one-third, one-half, two-thirds), some or all afrotherians have more adult-sized jaws (i.e., closer to 1.0 on the *y*-axis) than non-afrotherians. Afrotherians are shown in black, archontoglires in red, laurasiatheres in green, marsupials in brown. Abbreviations are as follows: Pc, *Procavia capensis*; ma, macroscelidids; ch, chrysochlorids; Pv, *Potamogale velox*; Ot, *Oryzorictes tetradactylus*; Ss, *Setifer setosus*; Te, *Tenrec ecaudatus*; st, strepsirhines; hy, hylobatids; Tj, *Tupaia javanica*; Sa, *Saimiri sp*.; Om, *Otocyon megalotis*; Er, *Erinaceus europaeus*; Eg, *Echinosorex gymnura*; Dm, *Didelphis marsupialis*. Rectangles above and diamonds below represent 95% confidence intervals of the mean. Non-overlapping polygons indicate significant difference at *p *= 0.05. Comparisons among high-level clades (Afrotheria, Archontoglires, Laurasiatheria) below exclude taxa (*Tenrec*, *Didelphis*) with CV of adult jaw length over 10.

This previously unquantified observation is consistent with the data collected here. In general, afrotherians without a full complement of permanent teeth are significantly larger (as a proportion of median adult jaw length) than non-afrotherians (such as didelphids, strepsirhines, hominoids, platyrrhines, scandentians, canids and erinaceids) at a comparable stage (Figure 2). Among the 22 genera sampled in this study (11 afrotherians, 4 strepsirhines, 2 gibbons, 1 tree shrew, 1 carnivoran, 2 erinaceids and 1 didelphid), all of the afrotherian genera exhibited specimens with 95% median adult jaw length that had less than 60% of their permanent, occluding premolars and molars erupted (Table 1). Stated differently, adult size among afrotherians is frequently reached prior to the eruption of many permanent teeth. Non-afrotherians showing less than 60% erupted cheek teeth were typically well under 90% median adult jaw length, except for one specimen of *Eulemur fulvus *(ZMB 4915) which reached 95% (Table 1). Two afrotherian groups (macroscelidids and chrysochlorids) had specimens that reached, or exceeded, median adult jaw length with less than 40% of their permanent cheek teeth erupted (Figure 2). Another index of skull size, condylobasal length, also shows that most afrotherians reach adult-proportions with fewer permanent cheek teeth erupted. However, because jaws are better represented in museum collections (particularly for the growth series of the taxa we sampled), we focus on the latter measurement in this paper.

**Table 1:** Minimum proportion of permanent cheek teeth erupted when 95% median adult jaw length reached (bold denotes afrotherians).

One-third erupted	**Chrysochloridae, Macroscelididae**
One-half erupted	***Potamogale*, *Oryzorictes*, *Setifer*, *Procavia*, *Tenrec*, **Eulemur
Two-thirds erupted	*Didelphis*, *Erinaceus*, *Echinosorex, Hylobates*, *Perodicticus*, *Lemur*, *Tupaia*
Fully erupted	*Otocyon*, *Otolemur*, *Symphalangus*

While some specimens of *Tenrec ecaudatus *follow this pattern, exceeding 95% median adult jaw length despite having incompletely erupted cheek teeth (Figure 1), this taxon shows a wide range of sizes throughout each eruption stage (Figure 2). Overall, *T. ecaudatus *does not reach a significantly larger proportion of median adult jaw length (and at one-third eruption of permanent cheek teeth is significantly smaller) than comparable stages of non-afrotherians. This results in part from the considerable variability in size within adult *T. ecaudatus*. With a coefficient of variation (CV) for jaw length over 17 across all adults (Table 2), *Tenrec *is nearly double that of the next-closest afrotherian (*Setifer*, CV = 9.8). Even controlling for sex, CV for adult jaw length in *Tenrec *still exceeds all others recorded in this study (male CV = 12.77; see Table 2). Its tendency for indeterminate growth with increasing age has been noted [25] and may actually comprise another possible similarity with otherwise morphologically disparate afrotherians, such as elephants [26]. While such large size in *Tenrec *is particularly noteworthy in zoo specimens, wild-caught individuals may also reach considerable size. For example, BMNH 97.9.1.56 (a male collected by Forsyth-Major from Vinanitelo, Madagascar) has a jaw measuring 83.3 mm from condyle to symphysis, a value about 20% longer than the median adult jaw length (including other adult males), and up to 33% longer than other specimens that show heavily worn teeth (e.g., ZMB 71582, jaw length 55.7 mm). Hence, the small ratios of juvenile to adult jaw length of some *Tenrec *specimens result in part from the influence of unusually large individuals on median adult jaw length, increasing the size of the denominator and decreasing the value of *Tenrec *ratios. A similar effect is evident in *Didelphis*, which also shows intra-specific dimorphism and has a large CV of adult jaw length (11.02, the only other taxon in this study to exceed 10). Importantly, however, no specimen of *Didelphis *comes within 90% of median adult jaw length without at least two-thirds of its cheek teeth erupted. In contrast, some *Tenrec *specimens approximate adult jaw length with under half of their permanent cheek teeth (Figure 1).

**Table 2:** Jaw length coefficient of variation (CV). Standard deviation expressed as a percentage of mean jaw length among specimens with all cheek teeth erupted; sample size (*n*) indicated in parentheses. Afrotherians are shown in bold.

Taxon (*n*)	CV jaw length
***Amblysomus hottentotus *(9)**	4.49
***Chrysochloris asiatica *(6)**	2.42
***Chrysospalax trevelyani *(9)**	2.08
***Eremitalpa granti *(12)**	2.42
***Elephantulus rozeti *(8)**	2.99
***Rhynchocyon sp*. (10)**	2.93
***Oryzorictes tetradactylus *(5)**	3.80
***Potamogale velox *(11)**	4.29
***Setifer setosus *(10)**	9.80
***Tenrec ecaudatus *(24)**	17.16 7.19 (4 females) 12.77 (7 males)
***Procavia capensis *(12)**	9.26
*Symphalangus syndactylus *(7)	4.03
*Hylobates sp*. (6)	7.21
*Saimiri sp*. (10)	5.50
*Otolemur crassicaudatus *(14)	5.30
*Perodicticus potto *(7)	4.67
*Lemur catta *(9)	3.15
*Eulemur fulvus *(8)	4.42
*Tupaia javanica *(10)	2.25
*Echinosorex gymnura *(13)	7.23
*Erinaceus sp*. (12)	4.73
*Otocyon megalotis *(10)	4.47
*Didelphis marsupialis *(9)	11.02

Despite these qualifications for *T. ecaudatus*, the afrotherians measured here with incompletely erupted permanent teeth, particularly sengis and golden moles, show adult proportions more frequently than non-afrotherians at a comparable stage of eruption (Table 1 and Figure 2). Non-afrotherians with unerupted cheek teeth are generally smaller than conspecifics with full, permanent dentitions. We interpret this to indicate that unlike afrotherians, most other mammals erupt the permanent dentition early enough in ontogeny so as to overlap extensively with growth.

### Absolute age at eruption of permanent cheek teeth

**Figure 3:**
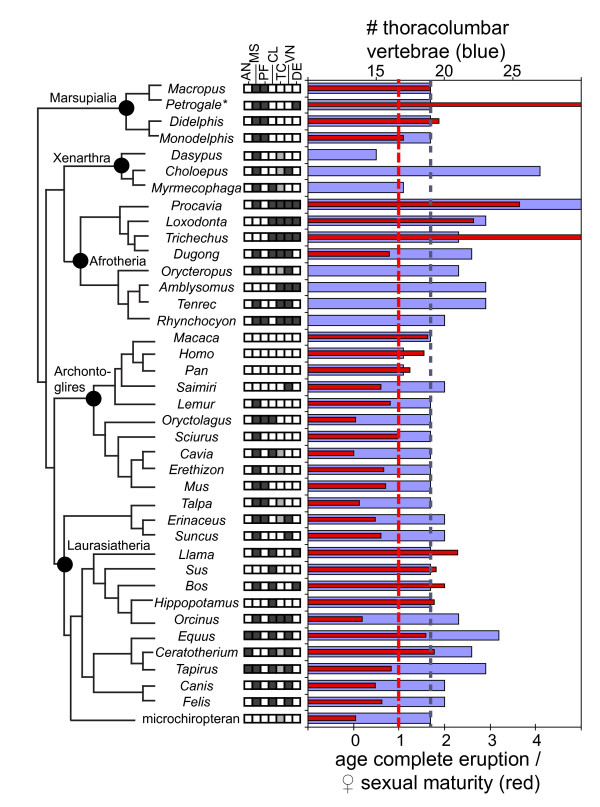
**Distribution of CCD-like characters on mammalian phylogeny**. Potential CCD-like features of Afrotheria optimized on a mammalian phylogeny [44]. Data on age at eruption are not yet known for small afrotherians (tenrecs, golden moles, sengis) or aardvarks. Based on the metric data presented in Figure 2 and in the text, we predict that when such data are available, age at complete cheek tooth eruption in small afrotherians will greatly postdate age at female sexual maturity (corresponding to long, horizontal red lines in elephants, hyraxes and manatees). Ages at completion of dental eruption in *Trichechus *and *Petrogale concinna *are arbitrarily set at the end of their recorded lifespan, reflecting the fact that these animals continually erupt supernumerary teeth. Dotted vertical lines represent 1.0 ratio of age at complete dental eruption/age at female sexual maturity (red, scale at bottom) and possession of 19 thoracolumbar vertebrae (blue, scale at top). Boxes at the center represent characters relating to phenotype observed in at least some afrotheres and human CCD patients, as follows: AN, unfused and projecting anterior nasal (black); MS, unfused metopic suture (black); PF, palatal fenestrae (black); CL, absent clavicles (black); TC, non-descended testicles (black), abdominal and ascrotal testicles (gray), scrotal testicles (white); VN, vertebral number (20 or more, black; 19 or less, white); DE, delayed eruption (after twice age at female sexual maturity, black; at or near female maturity, white). Scandentia, Dermoptera and Pholidota are not included owing to lack of data on dental ontogeny. Ages at female sexual maturity are taken from [45] except where indicated in Additional file 1. Vertebral data for sea cows represent averages from [49]. *Petrogale *is coded based on *P. concinna *for age at complete dental eruption and *P. penicillata *for thoracolumbar vertebral number owing to the lack of intact skeletons of the former in available collections.

For hyraxes, elephants, sea cows and many other mammals, absolute age at eruption of the complete, permanent dentition is already known. Figure 3 summarizes these data, standardized by age at female sexual maturity across mammals. Both sexual maturity and alternative scaling factors such as longevity may be skewed by outliers, such as unusually long-lived zoo specimens. Because data on sexual maturity are more widely available for wild populations than data on longevity, the former are used here.

Shigehara [15] noted that the eruption of the full permanent dentition may postdate sexual maturity in some mammals, including most old world primates, *Didelphis*, *Bos*, *Sus*, *Equus*, *Felis *and *Canis familiaris *(but not *C. lupus *or *C. mesomelas*). Among the taxa he studied, only perissodactyls showed completion of dental eruption after both sexual maturity and fusion of longbone epiphyses. However, some terrestrial artiodactyls show considerably later eruption of permanent cheek teeth than perissodactyls. In particular, *Hippopotamus *does not fully erupt all of its permanent cheek teeth until approximately 16 years of age [27]. The white rhinoceros shows the latest appearance of the complete permanent dentition among the perissodactyls sampled here, erupting all cheek teeth at around 8 years [28]. Some marsupials tend to show complete eruption of cheek teeth shortly after sexual maturity, a pattern greatly exaggerated in the dentally manatee-like wallaby *Petrogale concinna*, in which the seemingly endless eruption of supernumerary molars continues throughout life [14]. Hence, some artiodactyls, perissodactyls, carnivorans, anthropoid primates and marsupials show complete eruption of permanent cheek teeth at or near adulthood, ranging from slightly after to twice the age of female sexual maturity. Other non-afrotherian mammals show complete eruption prior to female sexual maturity (Figure 3).

The three afrotherians for which dental ontogeny is best known, *Loxodonta*, *Procavia *and *Trichechus*, show ages at complete cheek-tooth eruption that are, respectively, 2.6, 3.7 and more than 10 times the age of female sexual maturity. As mentioned above, *Trichechus *erupts an apparently unlimited number of cheek teeth throughout life [10]. In contrast, the highly-derived, peg-like cheek teeth of the sirenian *Dugong dugon *erupt between 6 and 9 years, at or shortly before sexual maturity at around 9 years; the prominent tusks of the dugong erupt later at around 15 years [29]. Compared with other major clades of mammals, and except for the cheek teeth of the dugong (but not its incisors), paenungulate afrotherians show late eruption of permanent teeth, postdating sexual maturity by a wide margin (Figure 3).

### CCD-like characters across mammals

In addition to humans, *Runx2 *sequence variation has also been documented in mice [30-32] and domestic dogs [33]. Homozygous mouse mutants lacking *Runx2 *fail to ossify membrane or endochondral bones [34]. These mutants exhibit initiation of tooth morphogenesis, but further development is incomplete [35]. Owing to an overall lack of bone development, they asphyxiate at birth from an unossified ribcage. Heterozygous mutant mice survive birth and show many of the same skeletodental anomalies as humans with CCD, including poor ossification of membrane bones such as the clavicle and elements of the braincase [34]. However, they develop relatively normal molars [32] possibly because mice have just a single dental generation, not two as in most other mammals; and in human CCD it is only the second generation of teeth that is affected [18,23]. Molars in mice and other mammals are commonly regarded among paleontologists as part of the same generation as permanent premolars, since both form the permanent dentition and partly overlap in terms of eruption. However, molars share a developmental origin from the primary dental lamina with 'first-generation' deciduous teeth, distal to the site of origin of the latter and buccal to the embryonic 'lingual successional lamina' (see [36], p. 186) which gives rise to permanent antemolars. Wang et al [31] observed that successionary cheek tooth buds, normally absent or very small in wild-type mice, were much more commonly observed in *Runx2 *mutant mice, further indicating that this locus may contribute to the morphogenetic pathway of the post-primary generation of teeth. They also noted greater expression of Runx2 protein in the lower than in the upper dentition. Our observations do not indicate different timing of eruption of the upper and lower teeth. Indeed, such a pattern would be problematic for mammals with functional occlusion. However, eruption of some lower tooth loci (e.g., p4) prior to their upper counterparts is not uncommon among mammals [37].

Sequences of afrotherian *Runx2 *available on public databases are limited, and at the time of writing include only partial sequences derived from ongoing genomes of *Loxodonta *and *Echinops *[38]. Only the former samples the DNA binding region, mutations to which may be linked to expression of CCD in humans [19]. Sequences of *Runx2 *from *Loxodonta *and other mammals such as *Pan*, *Macaca*, *Rattus*, *Mus*, *Equus *and *Canis *are easily alignable to human *Runx2 *for at least part of their length. These non-human mammals generally showed the conserved nucleotides present in the human wild type, not the mutations to *Runx2 *concentrated in the DNA binding domain among human CCD patients (see Tables 1 and 2 in [19]), except for a sequence of *Canis*. This specimen (GenBank XM_532158) [39] exhibits many changes to its *Runx2 *sequence, including a mutation commonly present among genetically documented human patients with CCD [19]: instead of the 'CGG' codon for arginine (R) at codon 225, they exhibit a 'CAG' codon for glutamine (Q). While hypodontia among domestic dogs (and other mammals) is known, they do not regularly show delayed eruption of the adult dentition (Figure 2).

**Figure 4:**
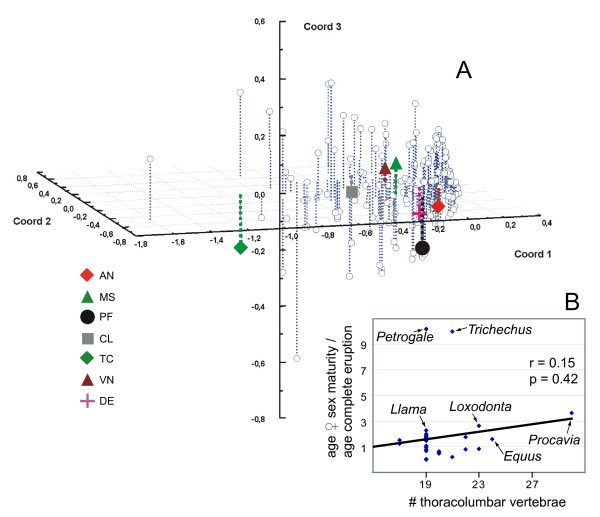
**Analyses of character correlation**. (A) Principal coordinate analysis based on morphological characters from [48], plus those identified in Figure 3. Coordinates 1, 2 and 3 explain 35.5%, 9.4% and 6.5% of variation, respectively (other axes do not exceed 5.2%). CCD-relevant characters are shown with polygons using abbreviations from Figure 3. (B) Correlation of number of thoracolumbar vertebrae with ratio of age at female sexual maturity to age at complete dental eruption. Line represents least squares fit and does not comprise a significant correlation, remaining insignificant with outliers (*Petrogale*, *Trichechus*, *Procavia*) removed. As in Figure 3, dental eruption for the macropodid *Petrogale *is based on *P. concinna*; vertebral number is based on *P. penicillata*.

At first glance the distribution of CCD-like characteristics across mammals, including delayed dental eruption, vertebral anomalies, clavicle reduction and testicondy (Figure 3), appears to show some co-occurrence. In particular, members of Afrotheria, Perissodactyla and terrestrial Cetartiodactyla lack clavicles and show some delay in the eruption of permanent teeth; and afrotheres and perissodactyls exhibit an increased number of thoracolumbar vertebrae. Examined quantitatively, however, and including other characters observed among CCD patients, this covariation is not significant (Figure 4). The distribution of these characters on a plot of the three principal coordinates that explain just over half of the variation does not place them in close proximity (Figure 4A), as would be expected if these features consistently occurred together (see Figure 1S in [40]). Nor is there a significant correlation between vertebral number and delay in eruption (Figure 4B). Whether or not this signal changes with consideration of a more complete morphological dataset, including ontogenetic data for small afrotheres as well as their other recently identified synapomorphies [3,6,7], remains to be seen.

## Discussion and conclusion

### Ontogeny of dental eruption

Based on these observations, we conclude that Leche was correct in his assessment that like large afrotherians in which dental ontogeny is well documented, sengis, tenrecs and golden moles also show relatively late eruption of the permanent dentition. Compared to non-afrotherians, permanent cheek teeth finish erupting only after these taxa have reached adult body size. Considered in isolation, this could mean that growth occurs faster and earlier in these afrotherians than other mammals, rather than the converse of late-erupting permanent teeth. However, although some afrotherians have relatively and/or absolutely long gestation times (e.g., hyraxes and elephants), which could be interpreted to support the hypothesis that growth takes place early, this is not true of Afrotheria as a whole. *Tenrec*, for example, has a shorter gestation time than *Cavia *(Rodentia) and *Saimiri *(Primates), although it can reach a larger body size than either taxon (Additional file 1). The sengi *Rhynchocyon *(with a mass of about 540 g and gestation of around 42 days) compares well with the rodent *Sciurus *(with a mass of about 530 g and gestation of around 44 days; see Additional file 1).

Notably, and in contrast to other afrotherians, the shrew-like tenrecid *Microgale *appears to erupt its molars (but not its premolars) relatively early during its development. Few museum collections have specimens of *Microgale *that exhibit fewer than three fully erupted molars [41]. In fact, the difficulty in distinguishing deciduous from permanent premolars in 'adult'-sized specimens of this taxon [8] has contributed to an unusually high level of species-level synonymy within this genus throughout much of the 20th century [41,42]. We assume that the frequent confusion of deciduous and permanent premolars in the literature results from the frequent retention of the former and late eruption of the latter, as in other small afrotherians examined here. In order to test this assumption, and include this taxon in our sample, we would need more sophisticated techniques to determine the deciduous versus permanent identity of premolars e.g., by using radiographs or computed tomography images, a sampling strategy that will be employed in a future study.

A further caveat is the dentition of the highly derived aardvark. Growth in this taxon is poorly known, and the last comprehensive summary of its dental development was published in 1934 (see [12]). From this we know that *Orycteropus *appears to lack functional deciduous teeth, although some may persist as rudiments in the gums well into adulthood. A summary in preparation (Lehmann, submitted) enables us to tentatively conclude that its permanent cheek teeth are erupted at a relatively early age, a result that is not surprising given its lack of functional deciduous teeth. Hence, as with the molar teeth of *Microgale *and *Dugong*, this too qualifies as an exception to what we believe otherwise to be a rule among afrotherians: that their permanent dentition finishes erupting relatively late during ontogeny, after sexual maturity and the occurrence of most growth.

### CCD character covariation

The apparent lack of character covariation (Figure 4) results in part from the fact that wild-type mammals represent ecologically viable populations, capable of successfully perpetuating their lineage, and should not be expected to mirror pathologies in a different species (*Homo sapiens*) with which they may share a developmental program. Regarding the genetic control of tooth succession, there is a complex interplay among genes expressing a number of morphogenic proteins, including Shh, BMP and FGF, which are mediated at least in part by *Runx2 *[18,30] and that relate to the development of teeth [30-32,35,43] and bone [20,23,34]. The pathologies of CCD-afflicted humans are a result of the decreased functionality of the DNA binding region in the regulatory locus *Runx2 *[19,20]. Depending on the extent of this decreased functionality, CCD humans show a mix of phenotypes, often (but not always) including features exhibited in afrotherians. Imperfect covariation among these characters among human CCD patients does not negate their relatively well-established link to *Runx2 *[19]. Similarly, imperfect correlation of CCD-like characters among mammals does not in itself negate the potential influence of *Runx2 *in their development.

Hence, a better understanding of *Runx2 *variation among afrotherians may yet provide insight into the mechanisms behind delayed eruption of the permanent teeth. We propose that this feature comprises an additional, shared characteristic of the newly recognized afrotherian clade.

## Methods

Crania housed in the Natural History Museum London (BMNH), Museum National d'Histoire Naturelle Paris (MNHN), Riksmuseet Stockholm (RS), University Museum of Zoology Cambridge (UMZC) and the Museum für Naturkunde Berlin (ZMB) were measured and examined for state of dental eruption (Additional file 2). Representatives of the four major placental clades as defined in [44], plus *Didelphis*, were chosen based on availability of good samples of juvenile specimens in the process of erupting cheek teeth. In order to minimize the confounding effects of intraspecific size variation (as with sexually dimorphic taxa), we focused on non-dimorphic species, with the exceptions of *Tenrec *and *Didelphis *owing to the obvious relevance of the former as an afrotherian, and owing to the accessibility of growth series for both taxa. Furthermore, we selected taxa that possess easily distinguishable deciduous and permanent cheek. Among afrotherians, this included tenrecs (*Oryzorictes tetradactylus*, *Potamogale velox*, *Setifer setosus *and *Tenrec ecaudatus*), chrysochlorids (*Amblysomus hottentotus*, *Chrysochloris asiatica*, *Chrysospalax trevelyani *and *Eremitalpa granti*), macroscelidids (*Rhynchocyon *[pooling *R. cirnei *and *R. petersi*] and *Petrodromus *['*Elephantulus*'] *rozeti*) and the hyrax *Procavia capensis*. Among archontoglires, we sampled *Lemur catta*, *Eulemur fulvus*, *Perodicticus potto*, *Otolemur crassicaudatus*, *Saimiri sciureus*, *Hylobates *(a pooled sample of *H. lar*, *H. muelleri *and *H. agilis*), *Symphalangus syndactylus *and *Tupaia javanica*. Among laurasiatheres, we sampled *Otocyon megalotis *(notable for its consistent expression of four molars), *Erinaceus europaeus *and *Echinosorex gymnura*. *Didelphis marsupialis *comprised the marsupial sample. The samples that pooled multiple species within a genus (*Rhynchocyon *and *Hylobates*) contain adults of a similar body size.

Degree of eruption was quantified in each specimen by expressing the number of permanent, occluding cheek teeth as a percentage of the norm for each species (Additional file 2). Specimens that have all normally occurring, permanent cheek teeth fully erupted are defined as 'adult'. For example, *Rhynchocyon *has three permanent premolars and two molars in the maxilla and dentary, yielding a total of 10 permanent upper and lower cheek teeth in each half of its dentition. Its deciduous premolars are also molariform, but are easily recognizable owing to morphology and wear relative to erupting, non-replaced molars. A specimen with only the first upper and lower molars erupted, and no permanent premolars, would have 2 out of 10, or 20%, of its permanent cheek teeth. For isolated specimens without associations, we assessed this percentage based on the material available (e.g., 1 out of 5 permanent cheek teeth of the dentary yields 20%). Only molariform premolars with an occlusive relationship between uppers and lowers were counted owing to the relative ease with which their deciduous and permanent generations can be distinguished. Recognizing tooth generation in some anterior cheek tooth loci in tenrecs and golden moles is particularly difficult [8,41]. For these taxa we defined a completely erupted, 'adult' dentition using the posterior premolars and molars identified in Additional file 2.

Data on age at complete permanent dentition, age at female sexual maturity, body mass and gestation were collected from [45] and the literature, as identified in Additional file 1.

PAST [46] and Statistica [47] were used for the principal coordinate analysis. Characters for thoracolumbar vertebrae (state 0 = 19 or less; state 1 = 20 or more) and eruption of the permanent dentition (state 0 = at/near sexual maturity; state 1 over twice the age of female sexual maturity) were added to a previously compiled morphological dataset [48], sampled here only for extant taxa. Missing data, inapplicables and polymorphisms in that matrix were replaced by entries representing the closest node with an unambiguously resolved character state, informed also by the fossil record. PAST was used to transform the matrix into pairwise distances between characters. These distances were then plotted with Statistica on axes representing the first three principal coordinates that explain over 50% of the variation (Figure 4A).

## Authors' contributions

RJA collected data, performed analyses and wrote the paper. TL collected data, performed analyses and contributed to writing the paper.

## Supplementary Material

Additional file 1 **Body size, gestation time and age at eruption and sexual maturity**. Data on life history variables. Click here for file

Additional file 2 **Metrics and other data for museum specimens**. Original measurements, museum numbers and proportion of permanent teeth erupted for each specimen. Click here for file

## References

[B1] Springer MS, Stanhope MJ, Madsen O, de Jong WW (2004). Molecules consolidate the placental mammal tree. Trends Ecol Evol.

[B2] Werdelin L, Nilsonne A (1999). The evolution of the scrotum and testicular descent in mammals: a phylogenetic view. J Theor Biol.

[B3] Mess A, Carter AM (2006). Evolutionary transformations in fetal membrane characters in Eutheria with special reference to Afrotheria. J Exp Zoolog B Mol Dev Evol.

[B4] Narita Y, Kuratani S (2005). Evolution of the vertebral formulae in mammals: a perspective on developmental constraints. J Exp Zoolog B Mol Dev Evol.

[B5] Sánchez-Villagra MR, Narita Y, Kuratani S (2007). Thoracolumbar vertebral number: the first skeletal synapomorphy for afrotherian mammals. System Biodivers.

[B6] Tabuce R, Marivaux L, Adaci M, Bensalah M, Hartenberger JL, Mahboubi M, Mebrouk F, Tafforeau P, Jaeger JJ (2007). Early Tertiary mammals from North Africa reinforce the molecular Afrotheria clade. Proc Roy Soc B.

[B7] Seiffert ER (2007). A new estimate of afrotherian phylogeny based on simultaneous analysis of genomic, morphological, and fossil evidence. BMC Evol Biol.

[B8] Leche W (1907). Zur Entwicklungsgeschichte des Zahnsystems der Säugetiere, zugleich ein Beitrag zur Stammengeschichte dieser Tiergruppe, Teil 2. Zool (Stuttgart).

[B9] Laws RM (1966). Age criteria for the African elephant, *Loxodonta africana*. East Afr Wildl J.

[B10] Domning DP, Hayek LAC (1984). Horizontal tooth replacement in the Amazonian manatee (*Trichechus inunguis*). Mammalia.

[B11] Steyn D, Hanks J (1983). Age determination and growth in the hyrax *Procavia capensis *(Mammalia: Procavidae). J Zool (Lond).

[B12] Anthony R (1934). La dentition de l'oryctérope, morphologie, développement, structure, interprétation. Ann Sci Naturel Zool.

[B13] van Nievelt AFH, Smith KK (2005). To replace or not to replace: the significance of reduced tooth replacement in marsupial and placental mammals. Paleobiology.

[B14] Sanson GD (1989). Morphological adaptations of teeth to diet and feeding in the Macropodoidea. Kangaroos, Wallabies and Rat-kangaroos.

[B15] Shigehara N (1980). Epiphyseal union, tooth eruption, and sexual maturation in the common tree shrew, with reference to its systematic problem. Primates.

[B16] Smith BH (2000). "Schultz's Rule" and the evolution of tooth emergence and replacement patterns in primates and ungulates. Development, Function and Evolution of Teeth.

[B17] Online Mendelian Inheritance in Man Database, entry #119600. http://www.ncbi.nlm.nih.gov/entrez/dispomim.cgi?id=119600.

[B18] Mundlos S, Otto F, Mundlos C, Mulliken JB, Aylsworth AS, Albright S, Lindhout D, Cole WG, Henn W, Knoll JHM, Owen MJ, Mertelsmann R, Zabel BU, Olsen BR (1997). Mutations involving the transcription factor CBFA1 cause cleidocranial dysplasia. Cell.

[B19] Otto F, Kanegane H, Mundlos S (2002). Mutations in the RUNX2 gene in patients with cleidocranial dysplasia. Hum Mutat.

[B20] Schroeder TM, Jensen ED, Westendorf JJ (2005). Runx2: a master organizer of gene transcription in developing and maturing osteoblasts. Birth Defects Res C Embryo Today.

[B21] Cogulu O, Munanoglu D, Karaca E, Onay H, Ozkinay F (2004). Cleidocranial dysplasia with new additional findings. Genet Couns.

[B22] Jensen BL, Kreiborg S (1990). Development of the dentition in cleidocranial dysplasia. J Oral Pathol Med.

[B23] Mundlos S (1999). Cleidocranial dysplasia: clinical and molecular genetics. J Med Genet.

[B24] Miles AEW, Grigson C (1990). Colyer's Variations and Diseases of the Teeth of Animals.

[B25] Eisenberg JF, Gould E (1970). The tenrecs: a study in mammalian behavior and evolution. Smithson Contrib Zool.

[B26] Shrader AM, Ferreira SM, McElveen ME, Lee PC, Moss CJ, vanAarde RJ (2006). Growth and age determination of African savanna elephants. J Zool (Lond).

[B27] Laws RM (1968). Dentition and ageing of the hippopotamus. East Afr Wildl J.

[B28] Hillman Smith K, Owen-Smith RN, Anderson JL, Hall Martin AJ, Selaladi JP (1986). Age estimation of the white rhinoceros (*Ceratotherium simum*). J Zool (Lond).

[B29] Marsh H (1980). Age determination of the dugong in northern Australia and its biological implications. Growth in Odontocetes and Sirenians: Problems in Age Determination.

[B30] Aberg T, Wang XP, Kim JH, Yamashiro T, Bei M, Rice R, Ryoo HM, Thesleff I (2004). Runx2 mediates FGF signaling from epithelium to mesenchyme during tooth morphogenesis. Dev Biol.

[B31] Wang X-P, Åberg T, James MJ, Levanon D, Groner Y, Thesleff I (2005). Runx2 (Cbfa1) affects Shh signalling and prevents the budding of putative successional teeth in mouse embryos. J Dent Res.

[B32] Zou SJ, D'Souza RN, Ahlberg T, Bronckers AL (2003). Tooth eruption and cementum formation in the Runx2 Cbfa1 heterozygous mouse. Arch Oral Biol.

[B33] Fondon JW, Garner HR (2004). Molecular origins of rapid and continuous morphological evolution. Proc Natl Acad Sci USA.

[B34] Otto F, Thornell AP, Crompton T, Denzel A, Gilmour KC, Rosewell IR, Stamp GWH, Beddington RSP, Mundlos S, Olsen BR, Selby PB, Owen MJ (1997). Cbfa1, a candidate gene for cleidocranial dysplasia syndrome, is essential for osteoblast differentiation and bone development. Cell.

[B35] D'Souza RN, Aberg T, Gaikwad J, Cavender A, Owen M, Karsenty G, Thesleff I (1999). Cbfa1 is required for epithelial-mesenchymal interactions regulating tooth development in mice. Development.

[B36] Luckett WP (1993). An ontogenetic assessment of dental homologies in therian mammals. Mammal Phylogeny: Mesozoic Differentiation, Multituberculates, Monotremes, Early Therians, and Marsupials.

[B37] Shabestari L, Taylor GN, Angus W (1967). Dental eruption pattern of the beagle. J Dent Res.

[B38] Ensemble Database. http://www.ensembl.org.

[B39] Lindblad-Toh K, Wade CM, Mikkelsen TS, Karlsson EK, Jaffe DB, Kamal M, Clamp M, Chang JL, Kulbokas EJ, Zody MC, Mauceli E, Xie X, Breen M, Wayne RK, Ostrander EA, Ponting CP, Galibert F, Smith DR, DeJong PJ, Kirkness E, Alvarez P, Biagi T, Brockman W, Butler J, Chin CW, Cook A, Cuff J, Daly MJ, DeCaprio D, Gnerre S, Grabherr M, Kellis M, Kleber M, Bardeleben C, Goodstadt L, Heger A, Hitte C, Kim L, Koepfli KP, Parker HG, Pollinger JP, Searle SM, Sutter NB, Thomas R, Webber C, Baldwin J, Abebe A, Abouelleil A, Aftuck L, Ait-Zahra M, Aldredge T, Allen N, An P, Anderson S, Antoine C, Arachchi H, Aslam A, Ayotte L, Bachantsang P, Barry A, Bayul T, Benamara M, Berlin A, Bessette D, Blitshteyn B, Bloom T, Blye J, Boguslavskiy L, Bonnet C, Boukhgalter B, Brown A, Cahill P, Calixte N, Camarata J, Cheshatsang Y, Chu J, Citroen M, Collymore A, Cooke P, Dawoe T, Daza R, Decktor K, DeGray S, Dhargay N, Dooley K, Dooley K, Dorje P, Dorjee K, Dorris L, Duffey N, Dupes A, Egbiremolen O, Elong R, Falk J, Farina A, Faro S, Ferguson D, Ferreira P, Fisher S, FitzGerald M, Foley K, Foley C, Franke A, Friedrich D, Gage D, Garber M, Gearin G, Giannoukos G, Goode T, Goyette A, Graham J, Grandbois E, Gyaltsen K, Hafez N, Hagopian D, Hagos B, Hall J, Healy C, Hegarty R, Honan T, Horn A, Houde N, Hughes L, Hunnicutt L, Husby M, Jester B, Jones C, Kamat A, Kanga B, Kells C, Khazanovich D, Kieu AC, Kisner P, Kumar M, Lance K, Landers T, Lara M, Lee W, Leger JP, Lennon N, Leuper L, LeVine S, Liu J, Liu X, Lokyitsang Y, Lokyitsang T, Lui A, Macdonald J, Major J, Marabella R, Maru K, Matthews C, McDonough S, Mehta T, Meldrim J, Melnikov A, Meneus L, Mihalev A, Mihova T, Miller K, Mittelman R, Mlenga V, Mulrain L, Munson G, Navidi A, Naylor J, Nguyen T, Nguyen N, Nguyen C, Nguyen T, Nicol R, Norbu N, Norbu C, Novod N, Nyima T, Olandt P, O'Neill B, O'Neill K, Osman S, Oyono L, Patti C, Perrin D, Phunkhang P, Pierre F, Priest M, Rachupka A, Raghuraman S, Rameau R, Ray V, Raymond C, Rege F, Rise C, Rogers J, Rogov P, Sahalie J, Settipalli S, Sharpe T, Shea T, Sheehan M, Sherpa N, Shi J, Shih D, Sloan J, Smith C, Sparrow T, Stalker J, Stange-Thomann N, Stavropoulos S, Stone C, Stone S, Sykes S, Tchuinga P, Tenzing P, Tesfaye S, Thoulutsang D, Thoulutsang Y, Topham K, Topping I, Tsamla T, Vassiliev H, Venkataraman V, Vo A, Wangchuk T, Wangdi T, Weiand M, Wilkinson J, Wilson A, Yadav S, Yang S, Yang X, Young G, Yu Q, Zainoun J, Zembek L, Zimmer A, Lander ES (2005). Genome sequence, comparative analysis and haplotype structure of the domestic dog. Nature.

[B40] Kangas AT, Evans AR, Thesleff I, Jernvall J (2004). Nonindependence of mammalian dental characters. Nature.

[B41] MacPhee RDE (1987). The shrew tenrecs of Madagascar: systematic revision and Holocene distribution of *Microgale *(Tenrecidae, Insectivora). Am Mus Novit.

[B42] Jenkins PD, Goodman S, Benstead J (2003). *Microgale*, shrew tenrecs. The Natural History of Madagascar.

[B43] Jernvall J, Keranen SV, Thesleff I (2000). Evolutionary modification of development in mammalian teeth: quantifying gene expression patterns and topography. Proc Natl Acad Sci USA.

[B44] Springer MS, Murphy WJ (2007). Mammalian evolution and biomedicine: new views from phylogeny. Biol Rev Camb Philos Soc.

[B45] Human Ageing Genomics Resource. http://genomics.senescence.info.

[B46] Hammer Ø, Harper DAT, Ryan PD (2001). PAST: paleontological statistics software package for education and data analysis. Palaeontol Electronica.

[B47] StatSoft, Inc (2001). STATISTICA for Windows Version 6. http://www.statsoft.com.

[B48] Asher RJ (2007). A web-database of mammalian morphology and a reanalysis of placental phylogeny. BMC Evol Biol.

[B49] Buchholtz EA, Booth AC, Webbink KE (2007). Vertebral anatomy in the Florida manatee, *Trichechus manatus latirostris*: a developmental and evolutionary analysis. Anat Rec.

